# Underwater internal traction-assisted reopenable clip-over-the-line method for full-thickness defect closure in a gastric collapse

**DOI:** 10.1055/a-2760-9728

**Published:** 2026-01-08

**Authors:** Tatsuma Nomura, Ken Ichikawa, Tomohide Hatanaka, Morihito Setsuda, Takashi Hamada, Hiroshi Kaneko, Katsumi Mukai

**Affiliations:** 1 Department of Gastroenterology, Suzuka General Hospital, Suzuka, Japan; 2 Department of Endoscopy Center, Suzuka General Hospital, Suzuka, Japan; 3 Department of Surgery, Suzuka General Hospital, Suzuka, Japan

We previously described a full-thickness defect closure technique using a line and reopenable clips 1 . However, complete closure of large defects in a collapsed stomach remains difficult. Recently, internal traction-assisted closure methods have been proposed to overcome this challenge 2 . Here, we report the first case of gastric full-thickness defect closure using an internal traction-assisted reopenable clip-over-the-line method (IT-ROLM) performed underwater.

**Fig. 1:**
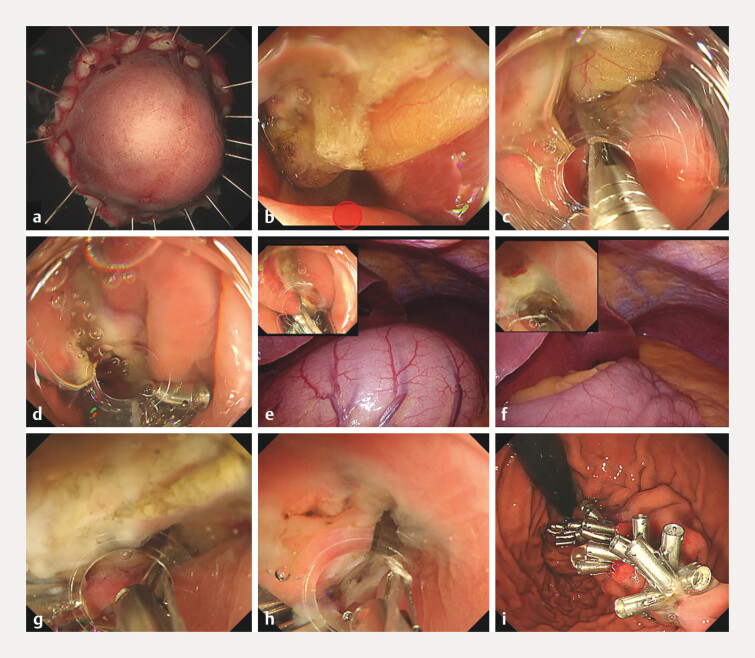
Gastric full-thickness defect closure in a gastric collapse situation using the underwater internal traction-assisted reopenable clip-over-the-line method. **a, b** A 40-mm full-thickness defect in the anterior wall of the upper gastric body under collapse conditions. The first clip with line was placed on the normal mucosa of the defect on the anal side (red circle). **c, d** The clip with a line through the tooth on one side was grasped at the greater curvature of the middle body. Continuous pulling of the line by the hand was used to extend the defect toward the anal side. **e, f** During abdominal observation, the stomach wall stretched when a small amount of gas insufflation was performed. Therefore, the stomach contents were replaced with a small amount of water under gas-free conditions using the gas-free immersion (GFI) system. **g, h** The defect edges were brought closer together for internal traction, into the appropriate position in underwater situation. This makes it easier to place the clip with the line through the tooth. **i** A full-thickness defect after complete closure via the internal traction-assisted reopenable clip-over-the-line method.

The patient had a 20-mm gastrointestinal stromal tumor in the upper gastric body ( Fig. 1 , Video 1 ). Therefore, we performed endoscopic full-thickness resection under laparoscopic assistance. First, two channels were used to control bleeding with dual-channel rapid hemostasis, and the mucosa and the muscle layer were incised 3 . After confirming that the subserosal tissue had been reached, a reopenable clip over the line traction was applied 4 . Strong traction was then applied to the tumor, and it was resected in approximately 25 minutes. The defect was completely closed using IT-ROLM. Initially, the first clip with line was fixed to the normal mucosa on the anal side of the defect. Another clip, with the line passed through one tooth, was placed on the greater curvature, providing traction toward the anal side (IT-ROLM).

Gastric full-thickness defect closure using the internal traction-assisted reopenable clip-over-the-line method in underwater situation.Video 1

When minimal gas insufflation was used for visualization, the gastric wall expanded; therefore, the stomach was carefully deaerated to create a low-pressure environment and then immersed in saline. The seal was maintained using a gas-free immersion system, which permits the infusion of small volumes of water into the hood 5 . It subsequently became easy to place clips at the edges of the defect under traction with the stomach deaerated. ROLM was repeated, and the defect was completely closed. The patient was discharged without adverse events.

Underwater IT-ROLM is a feasible and effective technique for closing large gastric full-thickness defects, particularly in a collapsed stomach.

Endoscopy_UCTN_Code_TTT_1AO_2AO
